# The Yin and Yang of Targeting KLRG1^+^ Tregs and Effector Cells

**DOI:** 10.3389/fimmu.2022.894508

**Published:** 2022-04-29

**Authors:** Samantha M. Borys, Arup K. Bag, Laurent Brossay, Dennis O. Adeegbe

**Affiliations:** ^1^Department of Molecular Microbiology and Immunology, Division of Biology and Medicine, Brown University Alpert Medical School, Providence, RI, United States; ^2^Department of Immunology, H. Lee Moffitt Cancer Center, Tampa, FL, United States

**Keywords:** regulatory T cells (T reg), cancer, KLRG1, immune modulation, Treg targeting

## Abstract

The literature surrounding KLRG1 has primarily focused on NK and CD8^+^ T cells. However, there is evidence that the most suppressive Tregs express KLRG1. Until now, the role of KLRG1 on Tregs has been mostly overlooked and remains to be elucidated. Here we review the current literature on KLRG1 with an emphasis on the KLRG1^+^ Treg subset role during cancer development and autoimmunity. KLRG1 has been recently proposed as a new checkpoint inhibitor target, but these studies focused on the effects of KLRG1 blockade on effector cells. We propose that when designing anti-tumor therapies targeting KLRG1, the effects on both effector cells and Tregs will have to be considered.

## Introduction

Killer cell lectin-like receptor G1 (KLRG1), originally termed mast cell function-associated antigen (MAFA) (1, 2), is expressed on subsets of NK and T cells in both mouse and human (3–6). KLRG1 binds E-, N-, and R- cadherin (7–9). Cadherins are widely expressed and involved in cell-to-cell adhesion, while cadherin loss is part of the epithelial mesenchymal transition (EMT) (10–14). Importantly, E-cadherin is also a ligand for the integrin α_E_(CD103)β7 (CD103) (15–17). However, the KLRG1 binding site to E-cadherin is distinct from the CD103 binding site (18). KLRG1 is a transmembrane glycoprotein with an extracellular C-type lectin-like domain and a cytoplasmic immunoreceptor tyrosine-based inhibitory motif (ITIM) (19). Phosphorylation of the ITIM tyrosine residue leads to recruitment of phosphatases SH2-containing inositol polyphosphate 5-phosphate (SHIP-1) and SH2-containing protein-tyrosine phosphatase 2 (SHP-2) (9, 20). The role of KLRG1 on effector NK and T cells has been well studied. However, KLRG1 is also expressed on a subset of CD4^+^Foxp3^+^ regulatory T cells (Treg) (21–23) and the role of KLRG1 on these cells is mostly unknown. Also unknown is the impact of targeting KLRG1 on the Treg response. Here we review the literature on the KLRG1^+^ Treg cell subset, its function, and the potential consequences of targeting this subset of cells.

## Inhibitory Role of KLRG1 in Effector Cells

It has been well established that KLRG1 is upregulated on highly differentiated NK and T cells, and KLRG1 signaling restrains their effector functions. High KLRG1 expression is associated with differentiation in both NK (4, 24) and T cells (25, 26) although it is not necessary for differentiation (27). KLRG1^+^CD8^+^ T cells were traditionally considered senescent (5, 28, 29). However, KLRG1^+^ effector CD8^+^ T cells display developmental plasticity and are able to down regulate KLRG1 (30). Additionally, KLRG1 engagement has been reported to inhibit AKT phosphorylation leading to proliferative dysfunction in T (31) and NK cells (32). In several studies KLRG1 upregulation has been observed on short-lived effector CD8^+^ T cells during viral and bacterial infection models, suggesting a role for KLRG1 in this context (5, 26, 33, 34).

A role for KLRG1 on CD4^+^ T cells during infection has also been documented. For instance, short lived, highly apoptotic, terminally differentiated and effector cytokine secreting KLRG1^+^CD4^+^ T cell populations are significantly upregulated in tuberculosis patients (35–39). Importantly, it has been shown that treatment with anti-KLRG1 blocking antibody enhances effector cytokine secretion and resolves disease severity (35). In addition, it was also shown that KLRG1^-/-^ mice display increased survivability after M. tuberculosis infection, largely due to an enhanced CD4^+^ T cell response (40). In support of these findings, it has been demonstrated that mucosal BCG vaccination, as well as the CAF01-adjuvanted fusion protein Ag85B-ESAT6, induces an antigen specific KLRG1^-^ CD4^+^ T cell population, which confers better protection against tuberculosis (41, 42). Another example is hepatitis B virus (HBV) vaccination, which was shown to be attenuated in patients infected with Hepatitis C virus (HCV). Interestingly, treatment with α-KLRG1 blocking antibody reverses the phenomena and improves the effectiveness of HBV vaccination in the setting of chronic HCV infection. Additionally, this study found that KLRG1 overexpression in CD4^+^ T cells impairs proliferation and secretion of IL-2 following TCR stimulation due to upregulation of cell cycle inhibitors p16^ink4a^ and p27^kip1^ and down regulation of AKT phosphorylation (43).

In addition to effector T and NK cells, KLRG1 has also been studied in the context of group 2 innate lymphoid cells (ILC2) (44). KLRG1 expression has been found on ILC2 of the lamina propria (45), lung (46, 47), and skin (48). KLRG1 interaction with E-cadherin has been shown to inhibit ILC2 proliferation and cytokine production (48), and when E-cadherin is downregulated, ILC2 cytokine production is unrestrained, leading to disease pathogenesis. Therefore, KLRG1 mediated inhibition of ILC2 *via* E-cadherin in certain tissues may limit the inflammatory response. However, KLRG1 is dispensable for ILC2 development and functions (47).

Regarding cancer, recent studies have demonstrated a role for KLRG1 on effector cells in anti-tumor immunity. Relevantly, a large number of studies have documented that downregulation of the KLRG1 ligand, E-cadherin, is associated with metastasis initiation (49–51). Some, but not all, of this association can be attributed to the role of cadherin in metastasis. EMT promotes metastasis and corresponds with loss of pro-adhesive E-cadherin and gain of expression of promigratory N-cadherin (14, 52, 53). However, this has been recently revisited as re-upregulation of E-cadherin appears to be required to seed in distant organs (54). E-cadherin transcripts have been reported in single cell RNA-seq datasets of melanoma, prostate, breast, HNSCC, and colorectal cancer cells (55, 56). Importantly, in several mouse tumor models, it was found that KLRG1 checkpoint blockade resulted in heightened anti-tumor immunity and better outcomes, while KLRG1/PD-1 combination therapy resulted in reduced primary tumor growth as well as metastasis development (55, 57). Notably, double blockade increased intratumoral T and NK cells, heightened T cell activation, and greater NK cell maturation (57). Altogether, these results suggest that targeting KLRG1 on effector cells might be beneficial to accelerate clearance of infection or cancer. However, the influence of KLRG1 on Tregs must be recognized and addressed for utmost therapeutic efficiency.

## Role of KLRG1 in Treg Cell Development and Homeostasis

The expression of KLRG1 is now well documented in NK cells, ILC2 and various T cell subsets including regulatory T cells (Treg) in mice (3, 6, 23, 28, 44, 58). Importantly, expression of KLRG1 on human Tregs has not been definitively substantiated. For instance, using single-cell ATAC-sequencing, a recent report showed that Tregs in blood, skin and fat tissues displayed accessibility at Treg signature genes including CTLA4 and CD39, but did not show accessibility in the KLRG1 locus, suggesting that KLRG1 in human Tregs may be silenced (59). Another group reported the absence of KLRG1 upregulation in effector Tregs in arthritis patients (60). In contrast, two papers reported the presence of a subset of Foxp3^+^ cells within KLRG1^+^CD4^+^ cells isolated from tuberculosis patient PBMC and ovarian cancer tumor microenvironment (35, 61). It is tempting to speculate that KLRG1 expression is tightly controlled in human Tregs, and its upregulation may depend on yet undefined molecular switches. Overall, additional studies exploring human Treg KLRG1 expression, especially in cancer settings, will need further examination. KLRG1 deficient animals develop without any evidence of abnormality in major organs or signs of autoimmunity/inflammatory disorders (27, 40, 62). Therefore, KLRG1 appears to be dispensable for natural Treg development and maintenance. The absence of KLRG1 expression in mature thymic CD4^+^ cells or in immature double-positive or double-negative thymocytes of young adult mice and humans indicates that the emergence of this marker on Tregs is a post thymic event (21, 22, 63). However, Tauro et al. have shown that a very small fraction of KLRG1^+^ Tregs are present in the thymocytes of young and adult mice (64). While not specifically addressed, it remains a possibility that these cells trafficked from peripheral tissues and re-entered the thymus, a retrograde movement pattern that can occur in peripheral T cells (65). In support of a post-thymic origin, KLRG1^+^ Tregs were found in peripheral lymphoid tissues of MHC class I and II deficient mice (21). In addition, it has been shown that KLRG1^+^ Tregs arise from a KLRG1^-^ subset during homeostasis and following antigenic experience (66, 67), which has been substantiated *via* fate mapping and RNA velocity analysis (58). Altogether, the data suggest that the majority of KLRG1 induction on Tregs is a post thymic phenomenon, and is likely due to exposure to antigen, inflammatory cues, or tissue specific reprogramming events.

## Possible Regulators of KLRG1 Expression on Tregs

Post thymic KLRG1 expression on Tregs can be controlled through various regulators under different conditions and likely plays an important role in tissue homeostasis. Several studies have indicated that KLRG1^+^ Tregs aggregate at sites of inflammation such as virally infected lungs, Peyer’s patches, and Lamina propria in the murine model of colitis or injured skeletal muscles (67–70). These findings suggest that inflammatory cytokines can induce KLRG1 expression on tissue resident Tregs. Recent studies about the role of specialized Tregs in tissue homeostasis revealed that the non-lymphoid tissue Tregs abundantly express α-chain of the cytokine receptor IL-33R (ST2) and GATA3. Additionally, these studies found that a large percentage of Tregs in ST2^+^ tissue express KLRG1, suggesting a possible regulatory role of IL-33 in ST2^+^KLRG1^+^ Tregs found in non-lymphoid tissue (69, 71, 72). However, using IL-33^-/-^ mice, it was demonstrated that IL-33 signaling through ST2 is not important for the development or maintenance of ST2^+^KLRG1^+^ Tregs (73). Instead, it appears that IL-33 acts as a cofactor of TGF-β-induced ST2^+^ Treg cells, which develop from naïve conventional T (Tconv) cells (69). Interestingly, IL-33 can induce IL-2 secretion by dendritic cells which specifically expand ST2^+^ Tregs (74). Although *in-vitro* TCR activation using anti-CD3/CD28 and IL-2 does not induce KLRG1 expression on Treg or Tconv (23, 25), it has been shown that extensive IL-2R signaling facilitated by IL-2, but not IL-15, is essential for the development of KLRG1^+^ Tregs *in vivo* (67). Additionally, Liu et al. has shown that protein O-GlcNAcylation dependent activation of IL-2/STAT5 signaling is requisite for the development of effector KLRG1^+^ Tregs during homeostasis (75). Similarly, Chinen et al. established the importance of activated STAT5 signaling in maintaining higher surface expression of KLRG1 in STAT5-CA^+^ (the constitutively active form of STAT5b) Tregs (76). In nonobese diabetic (NOD) mice and in Experimental Autoimmune Encephalomyelitis (EAE), chemical inhibition of CDK8/19 can induce the development of peripheral Tregs (pTreg) from antigen-stimulated effector, memory, or naïve CD4^+^ T cells, which express elevated KLRG1 and efficiently resolve disease severity. This suggests the possible connection of a CDK8/19 mediated STAT5 pathway in the emergence of KLRG1^+^ pTregs (77).

In addition to cytokine induced expression of KLRG1, multiple transcription regulators play a role in regulating KLRG1 expression. Lack of KLRG1 expression in Irf4^-/-^ Tregs has been reported, signifying that Irf4 may play an intrinsic role in KLRG1 expression and consequent differentiation of this Treg subpopulation (78). Another transcription factor, Bach2, which acts as a repressor of TCR-signaling induced IRF4-dependent differentiation of Treg cells, negatively regulates KLRG1 expression (79). Interestingly, E-protein (E2A/HEB) can directly bind to the promoter region of KLRG1 and negatively regulate its expression (80). Importantly, the KLRG1^+^ Treg subpopulation expresses very low levels of inhibitory DNA-binding/differentiation proteins (Id-3) and maintains a highly activated and suppressive state compared to the KLRG1^-^ population. In accordance with being a major regulator of T cell development and differentiation, Id-3 can also regulate KLRG1^+^ Treg development and functional properties (81). Similarly, the transcription factor Helios also acts as a negative regulator of KLRG1 expression on Tregs in chronic inflammatory settings (82). Notably, the transcription factor Myb also regulates the expression of KLRG1 on Tregs and maintains their differentiation during immune homeostasis (83). Finally, Cheng et al. determined that both KLRG1^-^ thymus derived and peripherally-induced Tregs can be converted into KLRG1^+^ Treg subsets (67). Collectively, these data suggest that microenvironmental cues contribute to the development of KLRG1 Treg subsets *in-vivo*.

## Association of KLRG1 Expression With Treg Cell Fate and Functions

KLRG1 expression on Tregs is associated with an activated and memory phenotype, and KLRG1^+^ Tregs express higher levels of CD69, CD103, CD25, Blimp-1, Foxp3 and CD62L than KLRG1^-^ counterparts. Lack of developmental heterogeneity, plasticity, and poor proliferative capability suggest that KLRG1^+^ Tregs are a terminally differentiated subpopulation (66, 67). This is supported by their decreased capability to expand and poor survivability following adoptive transfer into TCRα or IL-2Rβ deficient mice (21, 64, 66, 67). Interestingly, KLRG1^+^ Tregs also express higher levels of the suppressive molecules CTLA4 and CD39. Importantly, KLRG1^+^ Tregs have greater inhibition potential *in-vitro* than KLRG1^-^ counterparts, suggesting that KLRG1^+^ Tregs are a superior suppressive sub population (67, 84).

### KLRG1^+^ Treg and Autoimmunity

KLRG1^+^ Treg cells also feature prominently in numerous autoimmune diseases. For instance, the number of KLRG1^+^ Treg cells increases and positively correlates with disease severity of EAE while maintaining an activated and terminally differentiated short lived phenotype. These cells secrete high levels of IL-10 and IFN-γ and maintain their higher number through *in-situ* proliferation or conversion from a KLRG1^-^ Treg population (64). Similarly, KLRG1^+^ Treg populations presenting an effector memory-activated phenotype increase in the pancreas of NOD mice that model type 1 diabetes (T1D) (85, 86). Notably, non-classical CD4^+^CD49b^+^KLRG1^+^ Tregs have been shown to dampen arthritis severity in an IL-10-dependent but Foxp3-independent manner (87). Regarding graft versus host disease (GVHD), ST2^+^ Tregs ameliorate the intestinal damage through an IL-33 dependent increase of a KLRG1^+^ subpopulation (88). Finally, in a T-cell induced colitis model, it was revealed that Treg cells convert to KLRG1^+^ effector Tregs that express heightened αvβ8 integrin. In this context, the αvβ8 integrin mediated TGF-β pathway was shown to be essential, leading to high suppressive activity and inflammation control (89). Altogether, these reports highlight the therapeutic potential of KLRG1^+^ Tregs to prevent a variety of autoimmune diseases and GVHD.

### KLRG1^+^ Tregs and Cancer

Down regulation of E-cadherin or cadherin switching during EMT has been shown to promote specific accumulation and local expansion of KLRG1^+^GATA3^+^ Tregs. This is in part mediated through IL-33 secretion, which plays an important role in intestinal tumor progression (90). KLRG1^+^CD103^+^ Tregs may also play an important role in lung tumor progression. These cells accumulate in the tumor bearing lung tissue of mice and acquire the activation markers CD44, CD69, and the ectonucleotidase CD39 (91). Interestingly, Li et al. demonstrated that Treg specific ablation of the IL-33 receptor ST2 diminished the KLRG1^+^CD103^+^ Tregs, induced CD8^+^ T cell infiltration, and reduced the tumor burden, highlighting the dominant immunosuppressive and tumor promoting characteristics of this Treg subpopulation in lung cancer (92). In support of these findings, our lab also reported that infiltration of KLRG1^+^ Tregs directly correlates with tumor size. Specific depletion of KLRG1^+^ Tregs using JQ1/anti–PD-1 treatment increases the survivability of Kras^+/LSL-G12D^; Trp53^L/L^(KP) Non–Small Cell Lung Cancer bearing mice (84). Likewise, Kunisada et al. has shown that KLRG1^+^CD103^+^ Tregs also gather in subcutaneous models of MethA, MCA (fibrosarcoma), RLmale1 (radiation leukemia), and B16 melanoma (93). KLRG1^+^ Tregs have also been reported to amass in oncogene HPV16 E7-induced hyperproliferative premalignant skin lesions, indicating that KLRG1^+^ Tregs may regulate HPV-induced epithelial carcinoma (94). Similarly, an abundance of KLRG1^+^Foxp3^+^CD4^+^ T cells appear to accrue in the tumor microenvironment of ovarian and colon cancer, suggesting their role in impaired antitumor immunity (95). Altogether, the data support strategies aiming at targeting KLRG1^+^ Treg quantity and activity to improve anti-tumor immunity.

## Association of KLRG1 Expression With CD8^+^ Regulatory T Cells

In addition to CD4^+^Foxp3^+^ Treg cells, various types of CD8^+^ regulatory subpopulations (CD8^+^ Tregs) have been reported in different experimental systems (96). Among them, a CD44^+^CD122^+^Ly49^+^Helios^+^ subset has been shown to prevent autoimmunity by targeting CD4^+^ follicular T-helper cells *via* the non-classical molecule Qa-1 (96–100). Importantly, CD8^+^ Treg populations express only low levels of KLRG1 at the cell surface, suggesting that KLRG1 expression is likely to be differentially regulated on CD8^+^ Tregs and their CD4^+^ Treg counterparts (101–103). Mishra et al. has shown that TGF-β signal and Eomes are critical factors which maintain the regulatory phenotype and homeostasis of CD8^+^ Tregs expression leading to immune tolerance and autoimmunity prevention (104). Interestingly, Eomes expression in CD8^+^ Tregs inversely correlates with KLRG1 expression (104).

## Concluding Remarks

**Figure 1:**
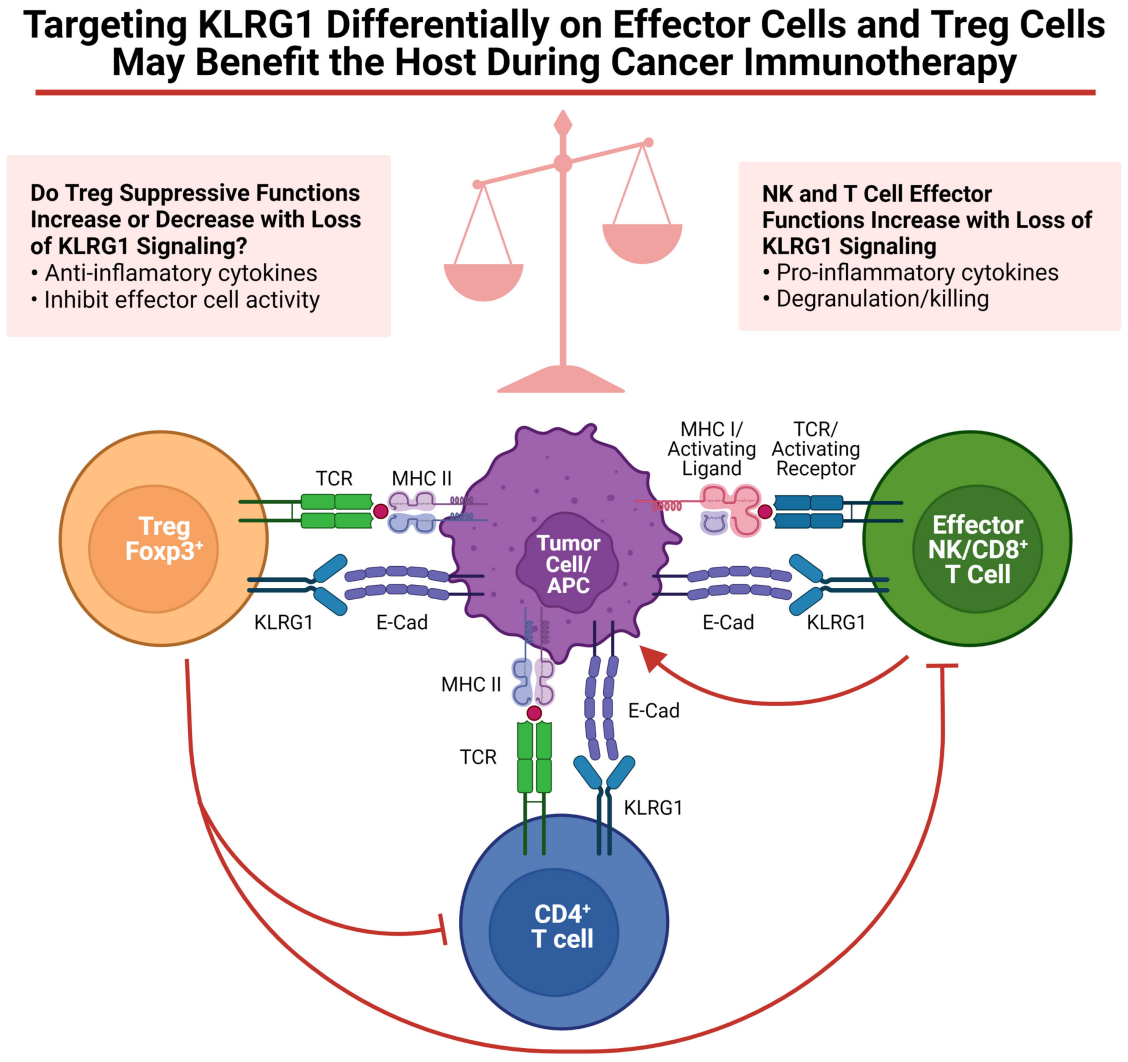
Targeting KLRG1 differentially on effector cells and Treg cells may benefit the host during cancer immunotherapy. Functions of KLRG1 signaling on various effector cells as well as Tregs are summarized. Figure was created on BioRender.

As discussed in this review, targeting KLRG1^+^ Treg cells to control tumor development, to prevent autoimmunity, or to control infectious diseases might benefit the host. However, as discussed above, Tregs are not the only cells expressing KLRG1; NK, ILC2 and T cells also express KLRG1. Regarding tumor development, enrichment of KLRG1^+^ T cells and NK cells in the tumor microenvironment has been observed in human tumors (55, 95). The role of cadherins on tumor immunosurveillance by KLRG1^+^ tumor infiltrating lymphocytes is just beginning to be investigated. Several labs have explored KLRG1 blockade as a possible immunotherapy (55, 57, 105). Although the focus of these studies was to unleash effector cells (i.e. NK cells and CD8^+^ T cells), it is possible that KLRG1^+^ Tregs were also targeted in these murine studies, potentially decreasing or increasing the benefit of this therapy (See **Figure 1**). Therefore, additional studies uncoupling the roles of KLRG1 on Tregs and NK cells are required. It has been clearly demonstrated that KLRG1 inhibits NK cell, CD8^+^ T cell and CD4^+^ T cell functions (4, 29, 31, 106, 107). However, it remains uncertain whether KLRG1 expression in mouse Tregs confers a distinctive module to their suppressive program. To specifically define the role of KLRG1 on NK cells or Tregs in a variety of murine tumor models, we have begun to cross KLRG1 floxed mice to Ncr-Cre and Foxp3-Cre respectively. Experiments with these mouse strains should help us to dissect the functional role of KLRG1 on these cells. Given the insufficient knowledge on KLRG1 expression in human Tregs, further studies will be necessary to validate the presence and role of these cells in various human pathological conditions including cancer. It is however tempting to speculate, as previously reported for CTLA-4 (108), that anti-tumor therapies aimed at unleashing KLRG1^+^ effector cells may target KLRG1^+^ Treg cells with an overall outcome dictated by the net effect on these two T cell populations. If KLRG1 expression on Tregs is validated in human cancers, it is reasonable to presume that KLRG1^+^ Tregs will be armed with a similar highly suppressive functional profile as in murine studies. In this regard, targeting KLRG1 signaling to differentially modulate KLRG1-expressing Tregs and effector cells for therapeutic outcomes will likely be a delicate balance (**Figure 1**).

## Author Contributions

AB, SB, LB, DA, wrote and edited the manuscript. All authors contributed to the article and approved the submitted version.

## Funding

This work was supported by NIH research grant R01 AI46709 (LB), Brown University Presidential Award (SB), and American Lung Association award ALA69-20210-02-01 (DA).

## Conflict of Interest

The authors declare that the research was conducted in the absence of any commercial or financial relationships that could be construed as a potential conflict of interest.

## Publisher’s Note

All claims expressed in this article are solely those of the authors and do not necessarily represent those of their affiliated organizations, or those of the publisher, the editors and the reviewers. Any product that may be evaluated in this article, or claim that may be made by its manufacturer, is not guaranteed or endorsed by the publisher.
